# Comparisons of Different Models on Dynamic Recrystallization of Plate during Asymmetrical Shear Rolling

**DOI:** 10.3390/ma11010151

**Published:** 2018-01-17

**Authors:** Tao Zhang, Lei Li, Shi-Hong Lu, Hai Gong, Yun-Xin Wu

**Affiliations:** 1 College of Mechanic and Electrical Engineering, Nanjing University of Aeronautics and Astronautics, Nanjing 210016, China; micronuaa@163.com (L.L.); nuaa297@163.com (S.-H.L.); 2 State Key Laboratory of High Performance Complex Manufacturing, Central South University, Changsha 410083, China; gonghai@csu.edu.cn (H.G.); yxwucsu@163.com (Y.-X.W.)

**Keywords:** asymmetrical shear rolling, microstructure evolution models, cellular automata, DRX behavior, grain refinement

## Abstract

Asymmetrical shear rolling with velocity asymmetry and geometry asymmetry is beneficial to enlarge deformation and refine grain size at the center of the thick plate compared to conventional symmetrical rolling. Dynamic recrystallization (DRX) plays a vital role in grain refinement during hot deformation. Finite element models (FEM) coupled with microstructure evolution models and cellular automata models (CA) are established to study the microstructure evolution of plate during asymmetrical shear rolling. The results show that a larger DRX fraction and a smaller average grain size can be obtained at the lower layer of the plate. The DRX fraction at the lower part increases with the ascending speed ratio, while that at upper part decreases. With the increase of the offset distance, the DRX fraction slightly decreases for the whole thickness of the plate. The differences in the DRX fraction and average grain size between the upper and lower surfaces increase with the ascending speed ratio; however, it varies little with the change of the speed ratio. Experiments are conducted and the CA models have a higher accuracy than FEM models as the grain morphology, DRX nuclei, and grain growth are taken into consideration in CA models, which are more similar to the actual DRX process during hot deformation.

## 1. Introduction

Aluminum alloy thick plates with light weight, high strength, good corrosion resistance, and formability, as a key material for integrated-structure parts, are widely used in the aerospace field [1]. Hot rolling is the key process of preparation for thick plates; however, the conventional symmetrical rolling process causes insufficient deformation and large grain at the center of the thick plate, resulting in poor mechanical properties at the center of the plate, which has become a barrier to the development of aerospace technologies. A new technique of asymmetrical shear rolling was adopted to solve uneven deformation [2].

Dynamic recrystallization (DRX) plays a vital role in grain refinement during hot rolling [3]. THe kinetic behavior of the DRX process under hot deformation for different materials had been studied by many researchers and each parameter in Avrami equation was fitted by experimental flow curves [4,5]. The modeling [6] and mechanism [7] of DRX behavior had been conducted by microstructure models and experiment. Maire et al. [8] established a full field modeling of dynamic and post-dynamic recrystallization and simulated topological evolutions by linking boundaries velocity and thermodynamic driving forces. Aashranth et al. [9] used an irreversible thermodynamics approach to identify stabilization stress and found that grain growth is the dominant microstructural phenomenon of DRX in the hot working process. Lin et al. [10] studied the effects of strain rate and deformation temperature on the microstructure evolution by integrating thermo-mechanical FEM with microstructure evolution models. In addition to the effects of strain rate and temperature, Wen et al. [11] analyzed the influences of the initial δ phase on DRX behavior and constructed DRX kinetics models by considering the synthetical influences of the initial δ phase. Microstructure variations due to the dynamic recrystallization (DRX) process have been analyzed by cellular automata (CA) methods by many researchers [12,13,14,15]. Lee et al. [16] and Seyed et al. [17] established a cellular automata model to analyze the microstructure variation of DRX during non-isothermal hot compression. Zheng et al. [18] established CA models to predict the microstructure variation of different recrystallization processes during multi-pass symmetrical rolling. Li et al. [19] and Jiang et al. [20] studied the relationship between microstructure and mechanical properties during asymmetrical rolling.

Asymmetrical shear rolling is a rolling method with velocity asymmetry and geometry asymmetry, which is beneficial to deformation permeation and grain refinement, especially at the center point of an ultra-thick plate. Many previous studies have focused on the effect of parameters of symmetrical rolling or different speed rolling on DRX fraction and grain size by microstructure evolution models or experiments. However, the effects of the speed ratio and offset distance on grain refinement or microstructure distribution of the plate in asymmetrical rolling are rarely published. Microstructure evolution models are empirical models and they are in relation to the variation of macro parameters in the rolling process, such as strain, strain rate, and temperature, in which the DRX process can be calculated very quickly. The nucleation and grain growth in the DRX process are considered in CA models and the topology of the microstructure can be obtained; however, the computing efficiency is relatively lower. Metallographic experiments can be conducted after rolling or just at given passes, but they are not able to track the microstructure variation during multi-pass rolling processes. Meanwhile, these experiments tend to damage the plate and cause material loss as well as decrease production efficiency. Therefore, the microstructure evolution in asymmetrical shear rolling as well as the effects of the speed ratio and offset distance on grain size variation need to be studied further in order to regulate and control the microstructure variation. However, the difference between these two methods is rarely studied and the accuracy of them needs to be compared in order to obtain the microstructure evolution efficiently. In this study, coupled FEM-microstructure evolution models and CA models are established to study the DRX behavior and variation of grain size for aluminum alloy plate during asymmetrical shear rolling, especially for the cross shear zone. The effects of the speed ratio and offset distance on the DRX fraction and average grain size are analyzed. Finally, experiments are conducted to compare the accuracy of these two kinds of models.

## 2. Asymmetrical Shear Rolling

**Figure 1:**
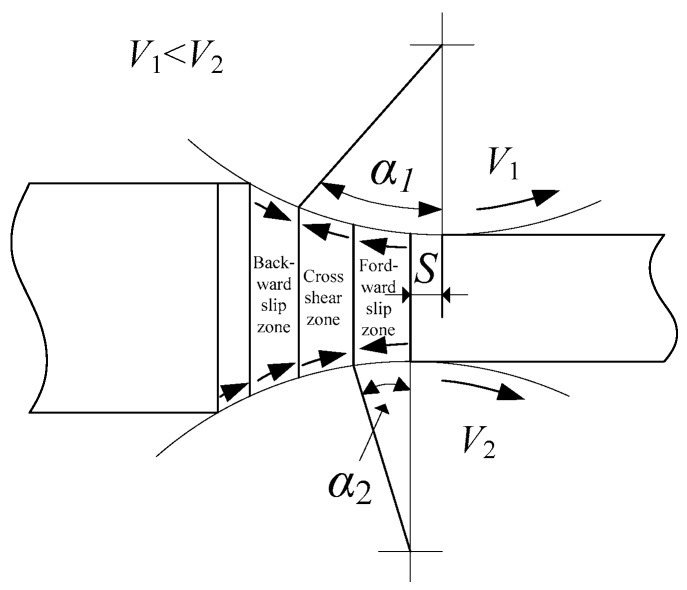
Schematic diagram of asymmetrical shear rolling (*V*_1_: Velocity of upper roll; *V*_2_: Velocity of lower roll; *S*: Offset distance; *α*_1_:Neutral angle of upper roll; *α*_2_:Neutral angle of lower roll).

Asymmetrical shear rolling is a rolling method with velocity asymmetry and geometry asymmetry. The lower roll has a larger velocity than the upper roll and there is also a horizontal offset distance (*S*) of the slow roll in outlet direction, as shown in Figure 1. As the lower work roll has a larger velocity, the neutral points of the upper and lower surfaces are not in the same vertical line. As a result, the deformation zone of the plate is divided into three zones: Backward slip zone, cross shear zone, and forward slip zone. The plate is subjected to strong shear strain in the cross shear zone, which is the prominent distinction to symmetrical rolling. The velocity asymmetry is beneficial for a large cross shear zone and strong shear strain at the center of thick plate, which contributes to deformation permeation into center part of the plate during the rolling process [21,22]. Due to velocity asymmetry, the plate will bend upwards for faster flow velocity of at the lower layer of the plate. The offset distance is adopted to apply a moment in the opposite direction of bending upwards to the plate during asymmetrical shear rolling. Through a good combination of speed ratio, offset distance, pass reduction, and initial thickness, quasi smooth plate can be obtained in asymmetrical shear rolling [23]. As a result, the plate can be subjected to strong shear strain with a small curvature, thus guaranteeing smooth multi-pass rolling deformation.

## 3. Models Establishment

### 3.1. Microstructure Evolution Model

The microstructure evolution model is a kind of empirical model to calculate the DRX fraction, dynamic recrystallized grain size, and average grain size during deformation, which is related to the strain, strain rate, and temperature. Therefore, an model is necessary to obtain the deformation and temperature distributions in the hot rolling process. In this study, single pass rolling models are established and rolling parameters are shown in Table 1. The material of the plate is 7055 aluminum alloy, and its constitutive equation [24] is shown in Equation (1). The initial rolling temperature is 410 °C and the inlet thickness is 250 mm. The heat transfer coefficient [25] to the environment is 5 W·m^2^·K^−1^ and the contact heat transfer coefficient to the work roll and emulsion is 30,000 W·m^2^·K^−1^.

(1) $$\dot{\varepsilon }=6.1192\times 10^{9}{\left[\sin h(0.0147\sigma )\right]}^{5.2212}\exp\left(\frac{-1.36382\times 10^{5}}{RT}\right)$$

where $\dot{\varepsilon }$ is strain rate, *σ* is flow stress, and *T* is temperature.

The DRX process begins when the strain of the material exceeds critical strain. In terms of micro perspective, the dislocation density is in relation to the strain and the DRX nuclei appear when reaching the critical dislocation density. Critical strain is connected with peak strain and they are both functions of temperature, strain, and strain rate in the models proposed by Sellars and Yada [26,27], as shown in Equations (2) and (3).

(2) $$\varepsilon_{p}=1.697\times 10^{-5}{\dot{\varepsilon }}^{0.15975}\exp(\frac{42448}{RT})$$

(3) $$\varepsilon_{c}=0.8\varepsilon_{p}$$

where *ε_p_* is peak strain, *ε_c_* is critical strain.

The DRX process mainly depends on two parameters: The DRX fraction *X_d_* and recrystallized grain size *D_d_*. In general, the value of the recrystallized grain size is much smaller than the initial grain size. Therefore, the initial grain can be intensively refined through sufficient DRX processing. The DRX fraction, recrystallized grain size, and average grain size can be calculated by Equations (4)–(7).

(4) $$X_{d}=1-\exp\left[-3.4\times 10^{-2}{\left(\frac{\varepsilon -\varepsilon_{c}}{\varepsilon_{0.5}}\right)}^{1.065}\right]$$

(5) $$\varepsilon_{0.5}=7.57\times 10^{-4}{\dot{\varepsilon }}^{0.09349}\exp\left(\frac{25707}{RT}\right)$$

(6) $$D_{d}=1.0473\times 10^{3}{\dot{\varepsilon }}^{0.129}\exp(\frac{-21542}{RT})$$

(7) $$\bar{D}=X_{d}D_{d}+(1-X_{d})D_{0}$$

where *X_d_* is the DRX fraction, *ε*_0.5_ is the strain corresponding to 50% DRX fraction, *D_d_* is the recrystallized grain size, *D*_0_ is the initial grain size, and $\bar{D}$ is the average grain size after deformation.

### 3.2. Cellular Automata Model

During the hot rolling process, work hardening and dynamic recovery are common phenomena; DRX will take place when the dislocation density reaches the critical value, which has a significant role in grain refinement. In this study, probabilistic CA models are adopted to predict the variation of the microstructure and grain size during hot rolling. In the CA model, the complex object is divided into cells discrete in time and space and each cell is defined by its dislocation density, crystal orientation, temperature, and strain rate, in which cells with the same crystal orientation belong to a specific grain. The status values of each cell are updated at each CA time step according to the transition rules between the cell and its neighborhood. In the CA model, the temperature, strain, strain rate, and flow stress are derived from FEM results. 

**Figure 2:**
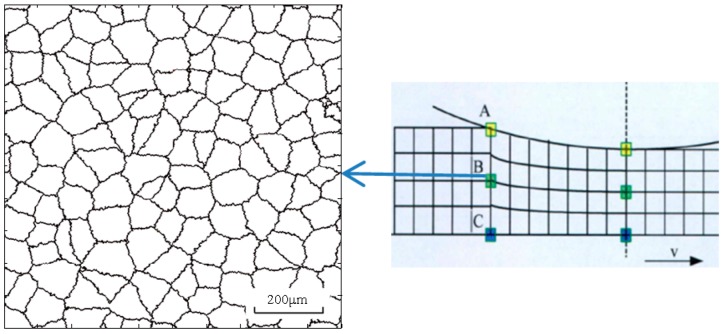
Initial microstructure of 7055 aluminum alloy (v represents rolling direction; A, B and C represent point at upper surface, center and lower point of the plate).

To acquire equiaxed grain and decrease microsegregation from casting, the homogenization treatment is conducted before hot rolling. The nucleation points are spread uniformly into the simulation zone and they grow into equiaxed grains in all directions with the same probability. Figure 2 shows the initial microstructure for the grain size of 100 μm, which corresponds to a point at the macro level in a finite element model.

7055 aluminum alloy is a kind of material with high stacking fault energy, which plays a vital role in its softening mechanism in hot deformation. The softening mechanism during hot rolling consists of two processes: Dnamic recovery and DRX. The flow stress decreases in the macro level and the dislocation density reduces in the micro level. The stacking fault energy of the material significantly affects the variation of dislocation density and thus influences the softening mechanism. The dislocation density in a material provides the relationship between flow stress at the macro level and microstructure characteristics at the micro level, as shown in Equation (8). Therefore, the variation of dislocation density can be obtained by true stress-strain curves [21]. A Kocks–Mecking (KM) model is used to describe the variation of dislocation density under different conditions [28], as shown in Equation (9).

(8) $$\sigma =\alpha \mu b\sqrt{\bar{\rho }}$$

(9) $$\frac{d\rho }{d\varepsilon }=k_{1}\sqrt{\rho }-k_{2}\rho$$

where $\bar{\rho }$ is the average dislocation density, *ρ* is the dislocation density, *k*_1_ and *k*_2_ are work hardening and dynamic softening coefficients, respectively; *α* is the dislocation interaction term, *μ* is the shear modulus, and *b* is the modulus of Burger’s vector.

Newly dynamically recrystallized nuclei start to appear when the dislocation density exceeds critical value. The nucleation rate ($\dot{n}$) can be calculated by models from Ding and Guo [29,30], as shown in Equation (10). The recrystallized nuclei start to grow into recrystallized grain after nucleation during the deformation process. The grain growth velocity (*v*) [31] is related to pressure (*p*) and the grain boundary (GB) mobility (*M*), as shown in Equation (11).

(10) $$\overset{}{\dot{n}}=C{\dot{\varepsilon }}^{m}\exp(\frac{-Q_{act}}{RT})$$

(11) v=Mp

where *C* is the material constant, *m* is the sensitivity coefficient of strain rate, and *Q_act_* is active energy.

## 4. Results and Discussion

### 4.1. DRX Fraction and Grain Size

**Figure 3:**
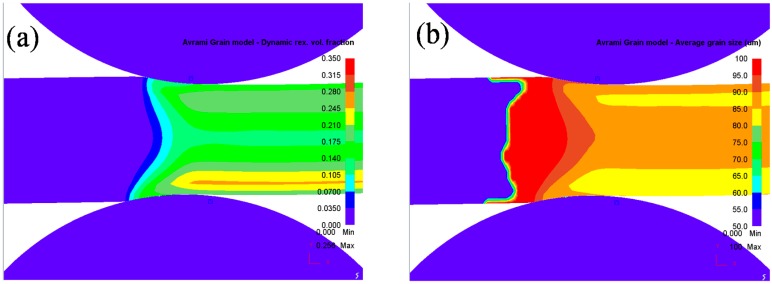
Distribution of (**a**) Dynamic recrystallization (DRX) fraction and (**b**) average grain size of plate during asymmetrical shear rolling.

**Figure 4:**
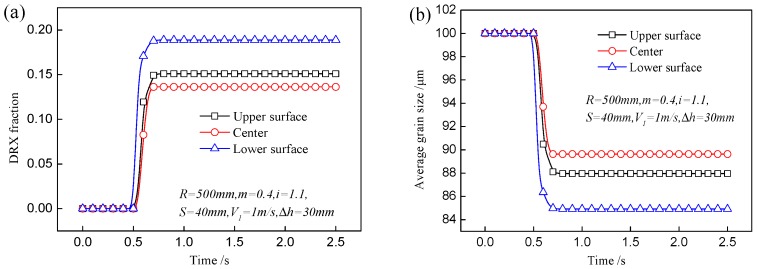
Variation of (**a**) DRX fraction and (**b**) average grain size at different positions of plate.

In the hot rolling process, DRX plays an important role in grain refinement. With velocity asymmetry and geometry asymmetry in asymmetrical shear rolling, the distributions of the DRX fraction and average grain size are asymmetrical, as shown in Figure 3. The DRX fraction at the lower layer of the plate is larger than that at the upper layer; as a result, finer grains appear at the lower layer. Due to the larger velocity of the lower work roll, the metal flow is faster and the equivalent strain is much larger than that at the upper layer; meanwhile, the temperature is higher than that at the upper layer because the contact time and heat exchange with the work roll are obviously reduced with a faster work velocity. However, the maximum DRX fraction and minimum average grain size are located subsurface of the plate (about 25 mm from surface). Due to direct contact with the work rolls, the metal flow at the surface is hindered by contact friction force and the equivalent strain is smaller than that at the subsurface. Therefore, large deformation and high temperature contribute to a more sufficient DRX process and intense grain refinement. Figure 4 shows variations of the DRX fraction and average grain size at different positions of the plate. The DRX fraction is 19.2% at the lower surface; it is 26% larger than that at the upper surface. For a given pass reduction, large deformation at the lower layer will result in small strain at the upper layer because the average deformation is constant. As a result, smaller grain size (84.5 μm) can be obtained at a lower surface compared to that (87.5 μm) at the upper surface.

In the author’s previous studies [21], the speed ratio and offset distance were found to significantly affect distributions of equivalent strain, shear strain, and temperature. According to Equations (4)–(7), the DRX fraction and average grain size are functions with strain, strain rate, and temperature. Therefore, the effects of the speed ratio and offset distance on the DRX process and grain size are necessarily analyzed to gain a better understanding of microstructure variation in asymmetrical rolling.

**Figure 5:**
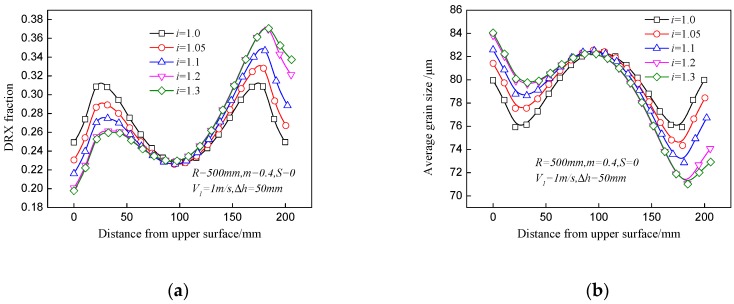
Effect of speed ratio on distributions of (**a**) DRX fraction and (**b**) average grain size in asymmetrical shear rolling.

Figure 5 shows the effect of speed ratio on distributions of the DRX fraction and average grain size in asymmetrical rolling. The DRX fractions at the lower surface and subsurface increase with ascending speed ratio; meanwhile, the average grain sizes at these positions decrease. However, a smaller DRX fraction and larger average grain size appear at the upper surface and subsurface with the increase of the speed ratio. As the speed ratio increases, the total strain and temperature are both enlarged at the lower part of the plate. Firstly, a large work roll velocity is beneficial to reduce the contact time and decrease the temperature drop, which results in higher temperature. Furthermore, higher temperature will reduce the flow stress of the plate, which has a positive effect on strain enlargement, and large deformation energy may in turn increase the temperature. With the increase of the speed ratio, the minimum DRX fraction moves to a position near to the upper part instead of locating at the center point of the plate, which illustrates that a large speed ratio is beneficial to strain permeation from the lower surface into upper part. The DRX fraction varies from 24.9% to 32.1% at the lower surface and the increase rate is 28.9%. 

**Figure 6:**
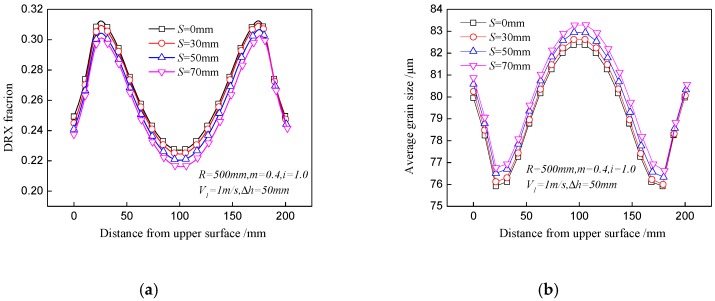
Effect of offset distance on distributions of (**a**) DRX fraction and (**b**) average grain size in asymmetrical shear rolling.

The effect of the speed ratio on distributions of the DRX fraction and average grain size is depicted in Figure 6. Increase of the offset distance will decrease the DRX fraction and increase the average grain size almost across the whole thickness of the plate. Although the arm of force between the two work rolls increases with the offset distance, the force value will decrease because the actual reduction is smaller than the given pass reduction. As a result, the deformation over the whole thickness of the plate will be decreased. The offset distance has little effect on temperature variation as the contact time of the upper surface and lower surface is almost the same. The DRX fraction at the center point decreases by only 1.1% when the offset distance varies from 0 to 70 mm, which indicates that the offset distance has slight effect on the DRX fraction and grain size.

**Figure 7:**
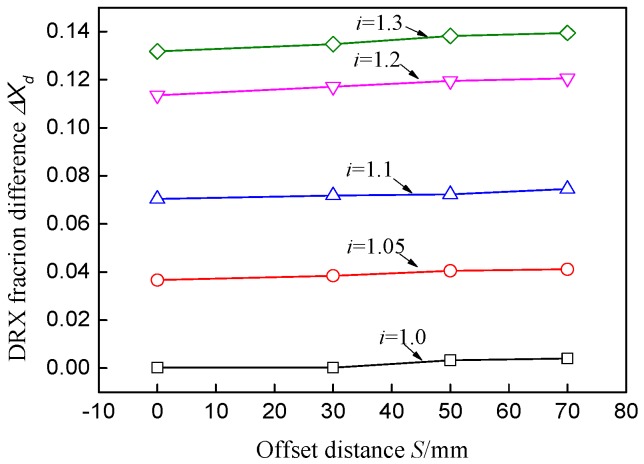
Effect of offset distance on DRX fraction difference between the upper and lower surfaces during asymmetrical shear rolling.

As discussed above, the speed ratio and offset distance have an effect on the asymmetrical distribution of the DRX fraction and average grain size between the upper and lower surfaces. In order to quantificationally describe this asymmetry, a parameter of the DRX fraction difference, Δ*X*, is defined. Figure 7 shows the effects of speed ratio and offset distance on Δ*X*, which increases significantly with the ascending speed ratio. A large speed ratio is beneficial to induce strong shear strain and will enlarge the strain gap between the upper and lower surfaces. The offset distance has little effect on the DRX fraction difference. Therefore, a proper speed ratio should be set by comprehensive consideration of a sufficient DRX fraction at the center point as well as a small DRX fraction difference between the upper and lower surfaces.

### 4.2. Microstructure Visualization

**Figure 8:**
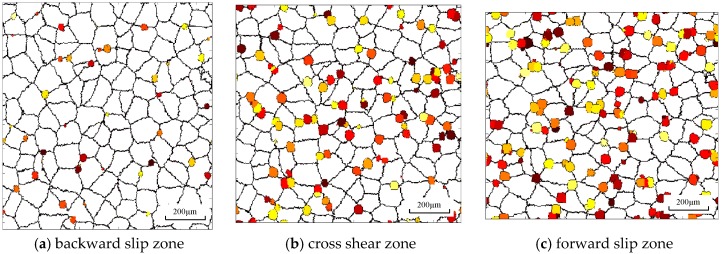
Microstructure under conditions *i* = 1.1 and *S* = 40 mm at the center point of the plate.

Figure 8 shows the microstructure variation at the center point during different deformation zones. The white region represents the matrix structure and colored grains represent DRX nuclei or DRX grains. It is obvious that dynamic nuclei first appear at grain boundaries for their high energy and then grow into recrystallized grains. In the backward slip zone, a few DRX nuclei are formed in the grain boundary and the grain shape is almost equiaxed because of small deformation. The recrystallization fraction and recrystallized grain size are increased sharply in the cross shear zone, as shown in Figure 8b. The large strain increases the deformation energy inside the material, which promotes the growth process of recrystallized grains. Furthermore, the temperature rises quickly in the cross shear zone due to the large heat energy induced by deformation energy, which enhances the thermally activated motion of atoms and molecules within the material. As a result, the recrystallization fraction changes from 1.8% in Figure 8a to 10% in Figure 8b and the average grain size reduces from 100 μm to 93.2 μm. In the forward slip zone, there is a slight increase of strain and recrystallization fraction.

**Figure 9:**
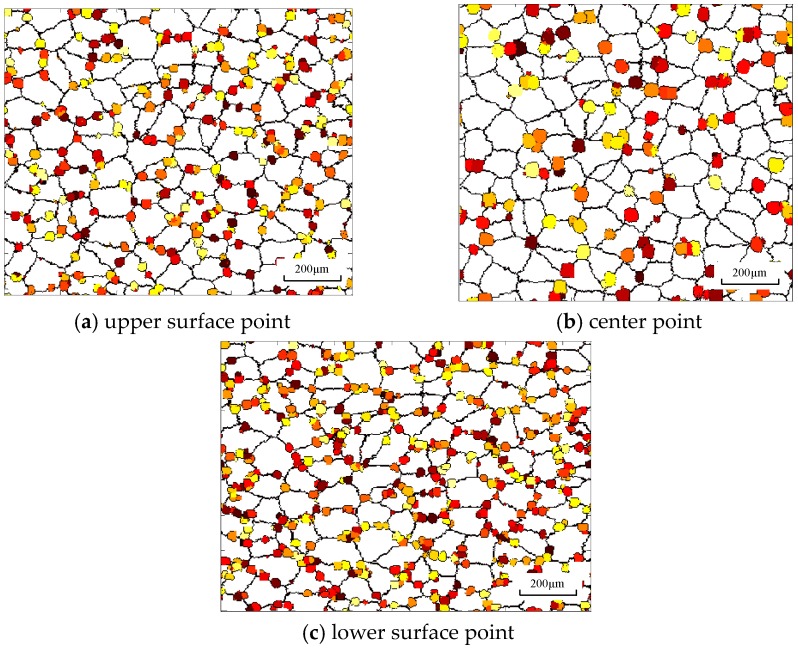
Microstructure under conditions *i* = 1.1 and *S* = 40 mm during the cross shear zone.

In asymmetrical shear rolling, asymmetrical strain distribution and temperature distribution will result in heterogeneous microstructure distribution. Figure 9 shows the microstructure variation at different positions in the cross shear zone. The grains at the surface point are elongated more seriously compared to the center point and the lower surface point is obviously subjected to the largest strain. Meanwhile, the number of dynamic nuclei at the surface is larger than that at the center because the large strain rate at the surface is beneficial to generate more nuclei, according to Equation (3). However, the recrystallized grain size is smaller than that at the center point. Due to the sufficient heat exchange with the work roll and emulsion, the temperature at the surface decreases sharply during rolling process, which hinders the grain growth; meanwhile, the temperature at the center is increased because of the thermal energy transferred from deformation energy. A lower temperature will increase the critical energy for the DRX process; as a result, despite the large strain, the recrystallization fraction of surface points is smaller than that of the center point. The recrystallization fraction is 20.1% at the center point, which is larger than that at the upper surface (13.2%) and lower surface (15.5%).

**Figure 10:**
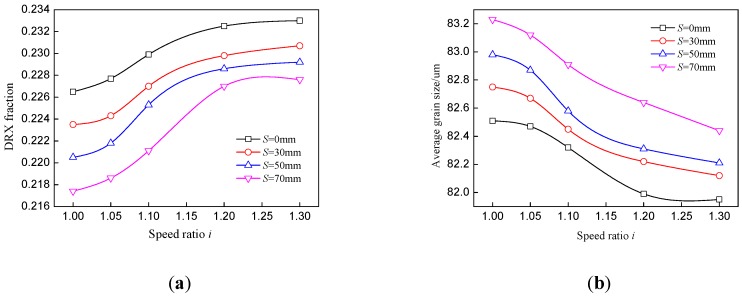
Effect of speed ratio on (**a**): Recrystallization fraction and (**b**): Average grain size at the center point of the plate.

**Figure 11:**
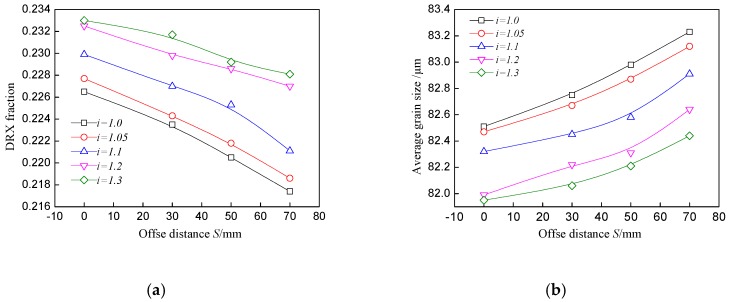
Effect of offset distance on (**a**): Recrystallization fraction and (**b**): Average grain size at the center point of the plate.

The movement asymmetry of the speed ratio and geometry asymmetry of the offset distance during asymmetrical shear rolling are the obvious distinction between symmetrical rolling. The effects of the speed ratio on the average grain size and recrystallization fraction at the center point are depicted in Figure 10. The recrystallization fraction increases sharply and the average grain size is refined remarkably when the speed ratio varies from 1.0 to 1.2. The strain and temperature both increase with the ascending speed ratio, which promotes the DRX process and grain refinement. However, the increase rate slows down by sequentially increasing the speed ratio (from 1.2 to 1.3), which indicates that a further increase of speed ratio has limited effect on grain refinement. The effect of the offset distance on the average grain size and recrystallization fraction is shown in Figure 11. With the increase of the offset distance, the recrystallization fraction decreases, while the average grain size increases. The actual pass reduction will decrease with the ascending offset distance, resulting in a smaller equivalent strain. The offset distance is mainly used to reduce the bending behavior of the plate in asymmetrical shear rolling. Therefore, a proper offset distance should be set by taking into consideration bending behavior and grain refinement.

## 5. Experiments

An asymmetrical shear rolling mill is remolded by a symmetrical rolling mill as follows: two work rolls with a diameter of 100 mm are driven by two motors to achieve a speed ratio (*i* = 1~4), while the velocity of the lower roll is fixed (0.0785 m/s). Different offset distances are achieved by inserting spacers with different thicknesses (0, 2, 4 mm) between bearing boxes of rolls and the frame. The material of the plate is 7055 aluminum alloy at casting state, with compositions of (wt %) 6.7Zn-2.6Mg-2.6Cu-0.15Fe-0.13Zr-0.12Si-0.06Ti, that is machined into plates with different sizes. The initial size of the plate is 150 mm (length) × 60 mm (width) × 20 mm (thickness). The initial rolling temperature is 410 °C and the plates are heated to the rolling temperature and then held for 30 min to acquire temperature balance in the heating furnace. The plates are immediately transferred to the rolling mill in less than 10 s and the heat exchange during the transfer period is disregarded. Due to the restriction of the mill capacity, each pass reduction is 2 mm and six rolling passes are conducted. A large speed ratio (*i* = 1.2) and large offset distance (*S* = 4 mm) are applied in the previous three rolling passes to acquire strong shear deformation. For the last three passes, the speed ratio turns to 1.1 and offset distance turns to 2 mm. 

**Figure 12:**
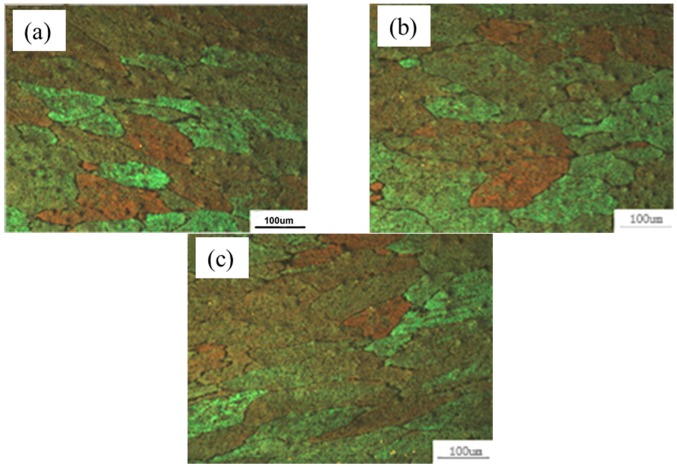
Experimental microstructures at different positions during asymmetrical shear rolling: (**a**) upper point; (**b**) center point; (**c**) lower point.

After multi-pass asymmetrical rolling, the specimens were cut at different positions (at the upper and lower surfaces as well as at the center) from the plates by a wire cutting machine. Then, the sections were polished by different types of abrasive paper and polishing paste, and etched by Keller’s reagent (2.5 mL HNO_3_ + 1.5 mL HCl + 1 mL HF + 95 mL H_2_O). The optical microstructures at different positions of the section plane were examined using a Leica DMI5000M image analyzer (Central South University, Changsha, China). The average single-circle-intercept grain sizes were measured using the method described in the American Society for Testing and Materials (ASTM) standards, which is beneficial for the grain size measurement of the material with obvious grain size difference at different positions. It should be guaranteed that there are at least 35 sections in each circle to improve the accuracy. Metallography experiments results are shown in Figure 12. A banded structure appears on the surface because of the large strain, and there is an angle of inclination of the banded structure due to the strong shear strain. The average grain sizes at the surface are smaller than that at the center point, and a lower point has better grain refinement compared to an upper point. Therefore, large strain and strong shear deformation is beneficial to grain refinement. Comparisons of experimental and simulated average grain size are shown in Table 2. The results simulated by the CA method have a higher accuracy than those of the FEM models. The DRX fraction and average grain size mainly depend on strain, strain rate, and temperature in the FEM model, which is an empirical model and is calculated by the differential method for different strains or temperatures. However, DRX consists of DRX nuclei and their growth, and the grain morphology will significantly affect the number of DRX nuclei and their growth process; as a result, the DRX fraction and average grain size is changed. The error of the FEM model is over 10%, while the maximum error of the CA model is 8.2%. 

## 6. Conclusions

(1) Microstructure evolution models and CA models are established to study the DRX behavior of the plate in asymmetrical shear rolling. The effects of the speed ratio and offset distance on the DRX fraction as well as grain size during asymmetrical shear rolling are analyzed.
(2) The distributions of the DRX fraction and average grain size are asymmetrical in asymmetrical shear rolling and the lower part of the plate has a larger DRX fraction and finer grain than the upper part. The DRX fraction difference between the upper and lower surfaces increases with ascending speed ratio, but the variation of the offset distance has little effect on this difference.
(3) More sufficient DRX and finer grain can be obtained with ascending speed ratio, while the increase of the offset distance will slightly decrease the DRX fraction and increase the average grain size.
(4) A proper speed ratio should be selected to ensure sufficient DRX on the basis of small bending behavior of the plate, and the existence of the offset distance is mainly used to decrease the bending behavior of the plate during asymmetrical shear rolling.
(5) CA models have a higher accuracy than FEM models because they take comprehensive consideration of DRX nuclei, grain growth, and grain morphology.

## Figures and Tables

**Table 1:** Single rolling parameters.

Parameter	Value
Work roll radius, *R*/mm	500
Shear friction coefficient, *m*	0.4
Velocity of lower roll, *V*_2_/(m/s)	1~1.3
Velocity of upper roll, *V*_1_/(m/s)	1
Pass reduction, Δ*h*/mm	30, 50
Speed ratio, *i* = *V*_2_/*V*_1_	1.00~1.3
Offset distance, *S*/mm	0~70

**Table 2:** Comparisons of simulated and experimental average grain size in asymmetrical shear rolling.

Average Grain Size	Upper Point	Center Point	Lower Point
Experimental value/μm	36.4	39.1	33.1
CA results/μm	38.5	40.7	35.8
Error (CA)	5.8%	4.1%	8.2%
FEM results/μm	40.2	44.8	36.4
Error (FEM)	10.4%	14.6%	10%
